# Pharmacological targets for the induction of ferroptosis: Focus on Neuroblastoma and Glioblastoma

**DOI:** 10.3389/fonc.2022.858480

**Published:** 2022-06-23

**Authors:** Luciano Ferrada, María José Barahona, Katterine Salazar, Alejandro S. Godoy, Matias Vera, Francisco Nualart

**Affiliations:** ^1^ Center for Advanced Microscopy CMA BIO BIO, University of Concepción, Concepcion, Chile; ^2^ Laboratory of Neurobiology and Stem Cells NeuroCellT, Department of Cellular Biology, Faculty of Biological Sciences, University of Concepcion, Concepción, Chile; ^3^ Centro de Biología Celular y Biomedicina, Facultad de Medicina y Ciencia, Universidad San Sebastián, Santiago, Chile

**Keywords:** ferroptosis, cancer cell, brain tumors, GPX4, system x_c_^−^, lipid ROS, iron

## Abstract

Neuroblastomas are the main extracranial tumors that affect children, while glioblastomas are the most lethal brain tumors, with a median survival time of less than 12 months, and the prognosis of these tumors is poor due to multidrug resistance. Thus, the development of new therapies for the treatment of these types of tumors is urgently needed. In this context, a new type of cell death with strong antitumor potential, called ferroptosis, has recently been described. Ferroptosis is molecularly, morphologically and biochemically different from the other types of cell death described to date because it continues in the absence of classical effectors of apoptosis and does not require the necroptotic machinery. In contrast, ferroptosis has been defined as an iron-dependent form of cell death that is inhibited by glutathione peroxidase 4 (GPX4) activity. Interestingly, ferroptosis can be induced pharmacologically, with potential antitumor activity *in vivo* and eventual application prospects in translational medicine. Here, we summarize the main pathways of pharmacological ferroptosis induction in tumor cells known to date, along with the limitations of, perspectives on and possible applications of this in the treatment of these tumors.

## Introduction

**Figure 1:**
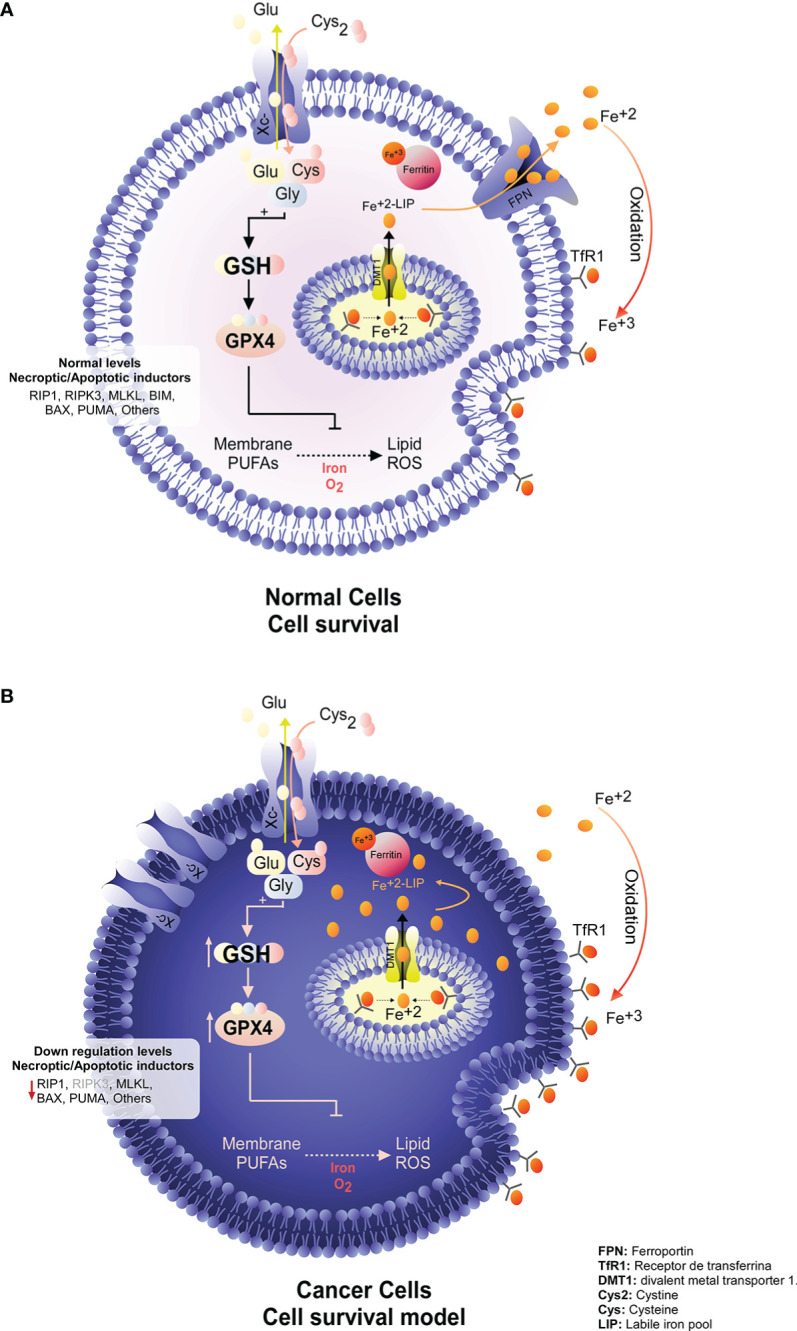
Survival programs in normal and tumor cells. **(A)** Under physiological conditions, normal cells maintain stable levels of death-executing proteins while maintaining a constant balance of nutrients and trace elements, promoting cell survival. **(B)** To avoid death, tumor cells activate various mechanisms, such as decreasing the expression of proapoptotic and necroptotic genes while increasing antioxidant defense by increasing GSH synthesis and GPX4 levels. In this way, ROS are efficiently eliminated, avoiding the damage produced by the accumulation of iron due to low FPN levels. This death evasion program makes many types of cancer highly difficult to treat, as classical apoptosis induction therapies fail because the machinery for the execution of this pathway is not available. FPN, Ferroportin; TfR1, Transferrin Receptor 1; DMT1, Divalent Metal Transporter 1; Cys2, Cystine; Cys, Cysteine; LIP, Labile Iron Pool.

Cancer is one of the most frequent pathologies worldwide; according to the World Health Organization (WHO) statistics, there were 18.1 million new cases and 9.6 million deaths related to this disease in 2018 (https://www.who.int/news-room/fact-sheets/detail/cancer). Cancers are difficult to treat because they employ multiple molecular mechanisms to evade different types of cell death, such as apoptosis, due to their overexpression of antiapoptotic proteins such as Bcl-2 and Bcl-xL and low expression of proapoptotic factors such as Bax, Bim and Puma (**Figures 1A, B**) (1). At the same time, it is known that the low efficacy of apoptosis induction with conventional therapies is due to the robust antioxidative defenses of tumor cells (2). Among the main antioxidants that confer apoptosis resistance on tumor cells is glutathione (GSH) (3, 4).

Due to the high resistance of tumors to apoptosis, the induction of necroptosis was postulated to be a potential therapeutic approach (5, 6). In contrast to apoptosis, which does not generate an inflammatory response, necroptosis induces death by cellular explosion, which generates a microenvironment of proinflammatory signals that could favor tumor death (5, 6). Thus, necroptosis, a form of regulated necrosis dependent on RIPK1, RIPK3 and MLKL, was postulated as a potential therapy for cancer (**Figures 1A, B**) (7–9). Unfortunately, several tumor cells evade necroptosis efficiently by inhibiting the expression of RIPK3 *via* epigenetic control mechanisms (10–12).

In line with this idea, new and emerging forms of regulated cell death with characteristics of necrotic disintegration have been described and postulated as treatments for cancer; among these, ferroptosis is highlighted (13–19). Here, we describe the main pharmacological targets for the induction of ferroptosis with emphasis on the treatment of brain tumors.

## Overview of the Induction of Ferroptosis in Cancer Cells: Targeting System $x_{\text{c}}^{-}$

**Figure 2:**
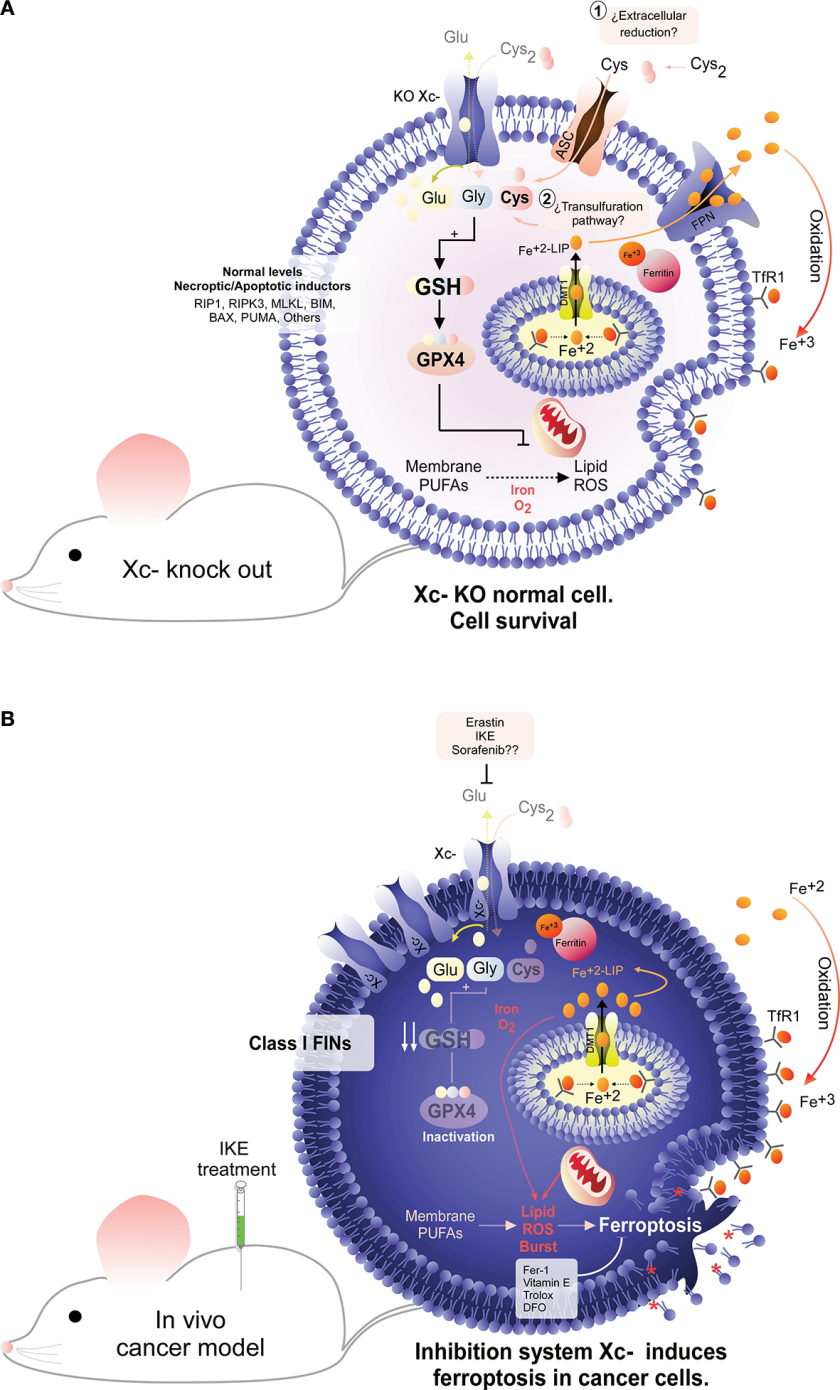
System $x_{\text{c}}^{-}$ dependence in cancer cells. Under physiological conditions, the nonessential amino acid cysteine ​​is present as cystine due to the extracellular oxidative environment. To maintain a stable intracellular cysteine ​​level, the presence of the cystine/glutamate antiporter (system $x_{\text{c}}^{-}$ ) is necessary. Interestingly, genetic deletion of system $x_{\text{c}}^{-}$ does not produce any damage in animals, suggesting that normal cells do not depend on this antiporter to maintain the intracellular cysteine ​​level. In line with this idea, compensatory mechanisms, such as the transsulfuration pathway, may exist for the recovery of the intracellular cysteine level **(A)**. Conversely, it has been widely described that tumor cells have a high dependence on system $x_{\text{c}}^{-}$ for the cellular uptake of cysteine ​​ **(B)**. Pharmacological inhibition of this antiporter results in the depletion of intracellular cysteine, inducing an abrupt decrease in the GSH level, which ultimately triggers inactivation of GPX4, the main hydroperoxidase in the cell. Inactivation of GPX4 due to inhibition of system $x_{\text{c}}^{-}$ results in an overwhelming overload of lipid ROS that ultimately induces tumor death by ferroptosis **(B)**. Interestingly, it has been determined that inhibition of system $x_{\text{c}}^{-}$ can induce tumor death both *in vitro* and *in vivo*, identifying this antiporter as a potential therapeutic target for cancer.

System $x_{\text{c}}^{-}$ is an antiporter that imports cystine and exports glutamate from the cell in a 1:1 ratio (**Figures 1A, B**). System $x_{\text{c}}^{-}$ is composed of 2 subunits: the SLC7A11 subunit (also called xCT), with a transport function and solute carrier family 3 member 2 (SLC3A2; also called CD98hc or 4F2hc), a chaperone with a plasma membrane anchoring function (20–23). For the purposes of this review, we refer only to the SLC7A11 subunit, given the importance of cystine transport to the cell (**Figures 1A, B**). In this context, the uptake of cystine into the cell is essential to maintain the redox state, since the reduced form of this amino acid (nonessential) is necessary for the biosynthesis of the main intracellular antioxidant, glutathione (GSH) (**Figure 1A**). Interestingly, most cancer cells overexpress SLC7A11 (22), suggesting a strong dependence on GSH to maintain the levels of controlled reactive oxygen species (ROS) (**Figure 1B**); thus, SLC7A11 is an potential therapeutic target. Interestingly, in 2012, it was determined that the small molecule erastin (13, 24) targeted SLC7A11 for inhibition, which led to depletion of GSH, inducing a type of death dependent on iron and lipid ROS, called ferroptosis (14). This type of cell death was inhibited by radical trapping antioxidants (RTAs) such as Ferrostatin-1 (Fer-1), lipophilic antioxidants such as vitamin E or iron chelators such as Desferoxamine (DFO) (18, 25), with potential application in the treatment of cancer and other pathologies (**Figure 2B**) (26).

Thus, when tumor cells are incubated with erastin, cell death is induced independent of caspases (13) or mitochondrial oxidative stress but in a manner dependent on iron, ROS and lipid ROS (14). Even though there is evidence that mitochondria could be involved, regulating the “avidity” for ferroptosis induction (27–29), they are not necessary for activation of this pathway (30). Inhibition of system $x_{\text{c}}^{-}$ results in intracellular depletion of cysteine ​​ because extracellular cystine (Cys_2_) is imported through SLC7A11 and reduced intracellularly to cysteine (**Figure 2B**) (16, 31). Intracellular cysteine ​​is necessary for the biosynthesis of GSH (16, 32). In turn, GSH is a cofactor for the selenoprotein GPX4, a hydroperoxidase responsible for detoxifying toxic hydroperoxides to alcohols (15). Therefore, erastin triggers indirect inhibition of GPX4 activity mediated by GSH depletion (**Figure 2B**).

Despite this apparent dependence of cells on system $x_{\text{c}}^{-}$ , animals with knockout of the *slc7a11* gene are fertile and develop completely normally (33), which prompted the consideration of SLC7A11 inhibition as an eventual cancer therapy with few adverse effects (**Figure 2A**).

**Figure 3:**
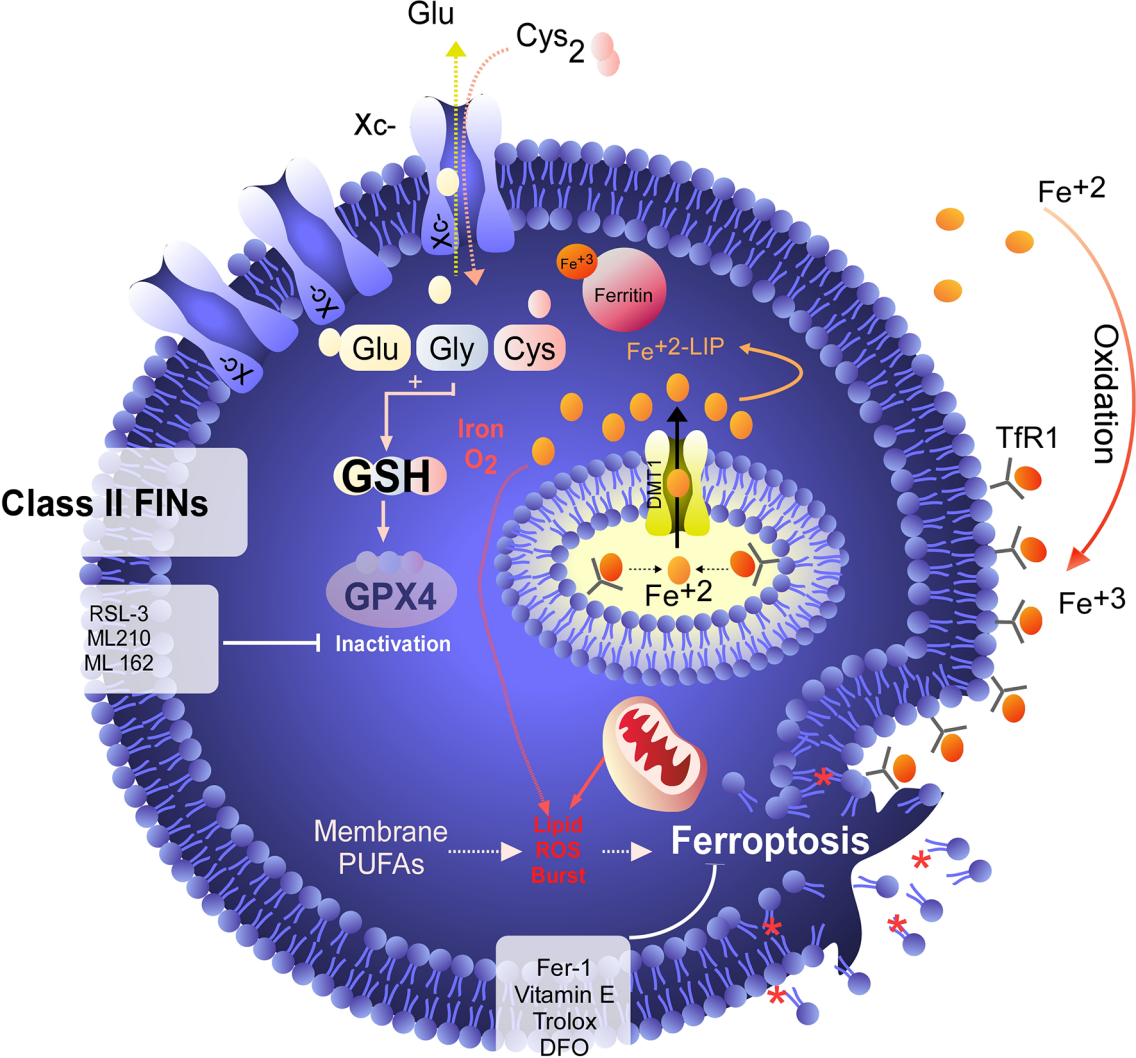
GPX4 as a target for ferroptosis induction. Unlike class I FINs, which indirectly inactivate GPX4, class II FINs such as RSL-3 directly inhibit GPX4, triggering ferroptosis independent of the GSH level. Direct inhibition of GPX4 results in rapid induction of ferroptosis, which can be inhibited by RTA or iron chelators. However, cell death is not inhibited by the recovery of cysteine uptake.

Thus, although many tumor cells can evade apoptosis and necroptosis due to their low expression of key genes for the activation of these pathways (**Figure 1B**) (1, 10, 11), RNA-seq data show that most cancer cells have high expression levels of SLC7A11 and GPX4 (https://portals.broadinstitute.org/ccle). Similarly, tumor cells are “addicted” to iron because they have decreased expression of ferroportin (FPN), the iron efflux pump, and overexpress the transferrin receptor (TfR1), the iron importer (**Figure 1B**) (34–37). Indeed, excess iron contributes to both tumor initiation and tumor growth (34). These observations indicate that SLC7A11, GPX4, iron and ferroptosis are potential therapeutic targets for cancer (**Figures 2B**, **3**). However, there are cancer cells that do not express FPN (MCF-7 cells, among others) and therefore accumulate excess intracellular iron but are still resistant to ferroptosis (38, 39). An explanation for this phenomenon is the recent finding that in addition to GPX4 and iron, acyl-CoA synthetase long-chain family member 4 (ACSL4) is another component that dictates sensitivity to ferroptosis (39). Reinforcing this concept, ACSL4 is a key protein because it incorporates long polyunsaturated fatty acids (PUFAs) into membranes, which allows lipid peroxidation to proceed and ferroptosis to be carried out (39–41). In another context, the erastin analog imidazole ketone erastin (IKE) has been shown to be metabolically stable and a potent inducer of ferroptosis in tumor cells *in vivo* (19, 42). Thus, induction of ferroptosis in tumor cells through inhibition of SLC7A11 may be a promising treatment for use in patients.

Interestingly, high doses of glutamate can inhibit system $x_{\text{c}}^{-}$ , emulating the effects induced by erastin (14, 43, 44). However, it is known that the responses to glutamate treatment are diverse and can induce cell death by apoptosis or necroptosis (45, 46) and eventually by other pathways of regulated necrosis. Thus, although high doses of glutamate can inhibit system $x_{\text{c}}^{-}$ , they are not necessarily a specific inducer of ferroptosis in tumor cells but could induce ferroptosis in normal tissues under pathophysiological conditions (44, 47–49).

### Ferroptosis Beyond the Inhibition of System $x_{\text{c}}^{-}$

**Figure 4:**
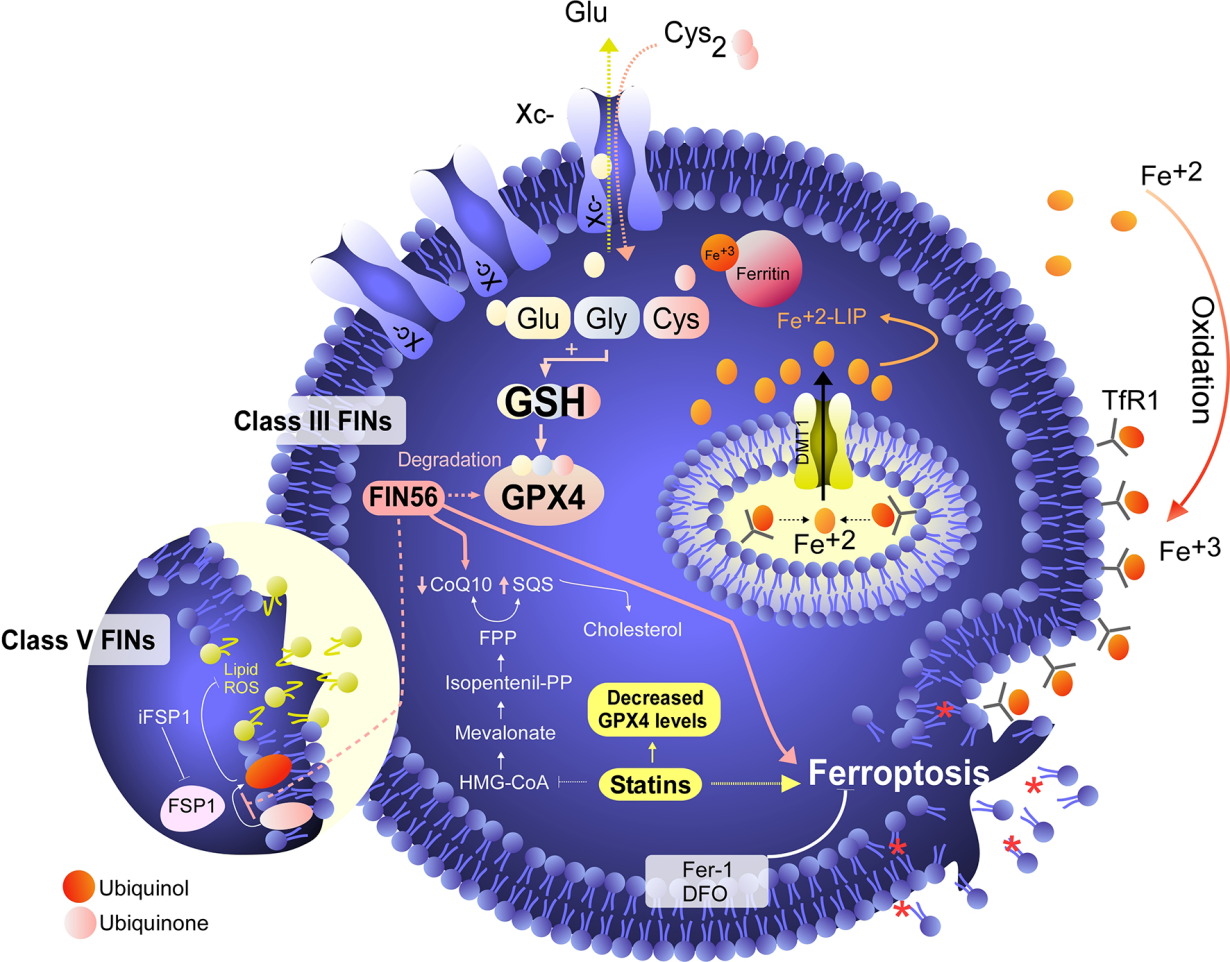
Degradation of GPX4/CoQ10 or inhibition of FSP1 induces ferroptosis in cancer cells. Class III FINs are molecules that act independently of system $x_{\text{c}}^{-}$ activity, the GSH level and direct inhibition of GPX4. These molecules, including FIN56, induce degradation of GPX4, which leads to ferroptosis induction. In addition to degrading GPX4, FIN56 also induces degradation of coenzyme Q10 (ubiquinone) by altering the mevalonate pathway. The importance of coenzyme Q10 degradation in the execution of ferroptosis is assumed because the function of a protein called FSP1 (a class V FIN) was recently described (33, 34). In this scenario, FSP1 converts extramitochondrial ubiquinone (the oxidized form of coenzyme Q10) to extramitochondrial ubiquinol (the reduced form of coenzyme Q10), and ubiquinol acts as an endogenous RTA that inhibits ferroptosis independent of the presence of GPX4. In this context, by inducing coenzyme Q10 degradation, FIN56 can inhibit the effects of FSP1 to confer resistance to ferroptosis. On the other hand, the inhibitor of FSP1 (iFSP1) controls ferroptosis without degrading CoQ10.

**Figure 5:**
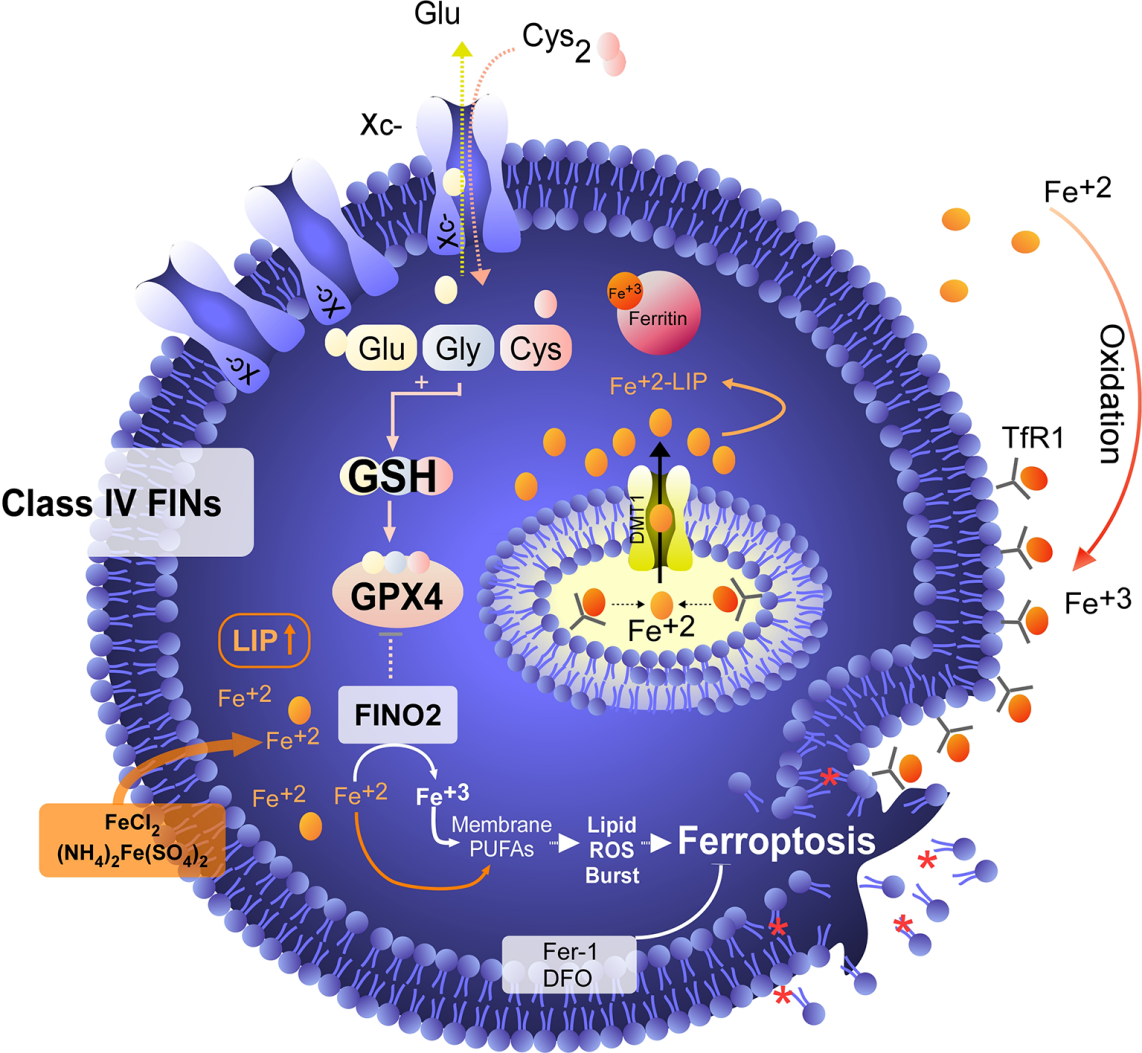
Iron overload or peroxidation induces ferroptosis in tumor cells. Class IV FINs are ferroptosis inducers that directly involve metabolism and iron levels in the cell. On the one hand, we have found synthetic molecules, such as FINO_2_, that alter the metabolism of iron, favoring its intracellular oxidation. In addition to promoting the oxidation of iron, FINO_2_ indirectly inhibits the activity of GPX4. On the other hand, when the labile iron pool (LIP) is increased by exogenous treatment with iron or iron nanoparticles, an overload of this metal is generated, which induces lipid peroxidation without the need for GPX4 inhibition. Class IV FINs are fairly attractive agents for the induction of ferroptosis because tumor cells are addicted to iron due to their low ferroportin (FPN) expression and high levels of transferrin receptor (TfR) expression, which favors an increase in the LIP. In this context, treatment with exogenous iron (e.g., FeCl_2_) in combination with FINO_2_ would eventually be a potent inducer of ferroptosis in tumor cells. Unfortunately, the development of FINO_2_ analogs for *in vivo* use is necessary to test whether the increases LIP and iron peroxidation are synergistic to specifically kill tumor cells.

Although the concept of ferroptosis was initially described in response to treatment with erastin, various ferroptosis inducers (FINs) have been developed to act independently of cystine uptake and GSH levels. FINs are currently classified into four classes (I-IV) (40, 50): class I FINs induce GSH depletion (**Figure 2B**), class II FINs inhibit GPX4 (**Figure 3**), class III FINs deplete GPX4 (**Figure 4**), and class IV FINs act through iron oxidation/iron overload (**Figure 5**) (summarized in **Table 1**). Interestingly, two research groups recently described a new player in the regulation of ferroptosis in parallel: ferroptosis suppressor protein 1 (FSP1) (**Figure 4**) (54, 55). Previously called apoptosis-inducing mitochondria-associated factor 2 (AIFM2), FSP1 is a flavoprotein with extramitochondrial oxidoreductase activity that can be recruited into the plasma membrane due to myristoylation. FSP1 in the plasma membrane catalyzes the conversion of ubiquinone (coenzyme Q10, CoQ10) to ubiquinol at the expense of NADPH (56).

**Table 1:** Principal Ferroptosis Inducers.

FIN Class	Target	Example
I	System $x_{\text{c}}^{-}$	Erastin, Sulfasalazine, Glutamate (14); IKE (19);
II	Inhibition of GPX4	RSL-3 (15); ML210, ML162 (51)
III	Depletion of GPX4	FIN56 (52), Statins (51), withaferin A (17)
IV	Oxidation/Overload of Iron	FINO_2_ (53); (NH4)_2_Fe(SO4)_2_ (17); FeCl_2_ (48)
V	Inhibition FSP1/Depletion CoQ10	iFSP1 (54); FIN56 (52)

Thus, the FSP1-ubiquinone-ubiquinol axis inhibits lipid peroxidation and ferroptosis in parallel to GPX4 and independent of GSH levels (54, 55). An inhibitor of FSP1 (iFSP1) (54) that may stimulate the induction of ferroptosis was identified by drug screening. In this context, the iFSP does not fit within any class of FINs (I-IV) because it does not target GPX4 or iron metabolism. Thus, we suggest that FINs that do not target GPX4 or iron metabolism but, as their mechanism involves CoQ10, can be classified into class V (**Figure 4**). Because FIN56 depletes GPX4 and CoQ10 (52), this compound has a dual classification and should also be reclassified into class V. Despite the existence of various ferroptosis inducers, not all of them have therapeutic potential *in vivo* (40). However, it has been shown that the use of class IV inducers could have potential therapeutic effects *in vivo* to treat high-risk neuroblastomas (17).

## Induction of Cell Death in Nervous Tissue

Normal adult neurons are equipped to survive because they express low levels of proapoptotic proteins and high levels of antiapoptotic proteins (57, 58). Furthermore, it has been shown that as neurons mature, they lose chemosensitivity to staurosporine and doxorubicin (58). This evidence suggests that brain tumors would be highly resistant to conventional antineoplastic agents, given the preconditioning of this type of cell to efficiently evade apoptosis. At the same time, it has been shown that tumor cells of astroglial origin (T98G, U251 and A172) efficiently evade necroptosis induced by chemotherapeutic agents because they do not express RIPK3 due to epigenetic modifications (10, 11).

Thus, the development of new therapies for the treatment of brain tumors that do not involve the induction of apoptosis or necroptosis as the main strategy is urgently needed. In this sense, in recent years, the induction of ferroptosis has gained great relevance as a possible therapeutic approach to induce cell death in brain tumors (17, 50, 59, 60). Considering this concept in the following sections, we focus on the induction of ferroptosis in neuroblastoma (NB) and glioblastoma multiforme (GBM).

### Neuroblastoma

NB is the most common pediatric extracranial tumor, accounting for more than 15% of all cancer deaths in children (61). NB is classified as low-, intermediate- and high-risk (62). While low-risk and intermediate-risk NBs generally have a good prognosis given that they develop into benign ganglioneuromas or enter remission due to surgical or pharmacological treatment, high-risk NBs have few treatment options (17, 62, 63). The main diagnostic characteristics of high-risk NB are that it appears after 18 months of age, has MYCN amplification, or exhibits activation of telomere maintenance mechanisms (62, 63). In line with this observation, current therapies against the NB include treatment with cycles of cisplatin, etoposide, vincristine, doxorubicin, and cyclophosphamide (64), which are preferential inducers of apoptosis. However, this type of pharmacological treatment generates multidrug-resistant clones, which greatly hinders the eradication of this type of tumor and favors its relapse (64).

### Classical Pharmacological Induction of Ferroptosis in Neuroblastoma

Considering that classical NB eradication therapies generally fail, it has been proposed that the induction of ferroptosis could be a feasible therapeutic approach. In this context, when the sensitivity of NB cell lines to classic ferroptosis inducers such as erastin or RSL-3 was studied (**Figures 2B**, **3**), it was determined that most of the models (SHSY-5Y, SK-N-SH, NB69, SK-N -DZ, NLF, and CHP-134 cells, among others) are highly insensitive to SLC7A11 or GPX4 inhibition (17, 65, 66). At the same time, there is very little information on the potential use of iFSP1 as a possible strategy against NB, since this compound has only been tested in the IMR-5/75 cell line without major effects on viability (54). Based on this background, the scientific community has focused on the search for new strategies for ferroptosis induction in NB, not through the classical targets but instead through the use of combined therapies or noncanonical inducers of ferroptosis, as potential treatments for high-risk NB (17, 67).

## Typical and Atypical Pathways to Induce Ferroptosis in Neuroblastoma

Because NB generally presents resistance to Erastin and RSL-3, it is necessary to search for new ferroptosis inducers. To this end, it was recently determined that treatment with the natural compound withaferin A (WA) can eradicate high-risk NB (17) by inducing ferroptosis through the canonical pathway, this means with GPX4 as a direct target. On the other hand, *via* a noncanonical pathway, where keap1 is the target, thus favoring an increase in labile iron pool (LIP) (17). This dual behavior of WA, similar to that of a mixture of FIN56 and FINO2 (**Figures 4**, **5**) (52, 53) (compounds that have not been tested in NB models), seems to render it a promising drug therapy for NB, since to date, it is unknown whether *in vivo* application of FINO_2_ is possible (40). Fortunately, WA has been shown to be effective in promoting the eradication of NB *in vivo* (17, 50). Interestingly, even though WA has been shown to induce iron-dependent lipid peroxidation and GPX4 depletion, Fer-1 treatment does not completely rescue NB cells from cell death (17). This suggests two alternatives; the first is that WA induces other types of ferroptosis-independent death in NB. However, WA induces lipid peroxidation, which is completely inhibited by treatment with DFO and partially inhibited by Fer-1, suggesting a strong iron dependence (17).

In this context, as a second alternative, the authors suggest that WA could eventually favor an overload of lipid ROS of various origins that may not necessarily be inhibited by Fer-1, such as lipid ROS generated by H_2_O_2_ (17). It is important to note that Fer-1 does not inhibit death induced by H_2_O_2_ treatment (14) or by extracellular H_2_O_2_ production mediated by pharmacological doses of ascorbic acid (68), because these treatments preferentially induce conventional necrosis. However, it has recently been determined that NADPH-cytochrome P450 reductase (POR) favors the induction of ferroptosis due to the cytoplasmic production of H_2_O_2_ (69), which is inhibited by Fer-1 or by the intracellular expression of catalase (69) but thus far, this finding is limited to cervical cancer cells (HeLa).

In this context, and considering the particularities of NB cells, it is likely that it is possible to classify the cell death induced in this type of tumor (or others) as ferroptosis, even when it is not inhibited by Fer-1, if it has other hallmarks, such as lipid peroxidation and iron dependence. Indeed, it has recently been determined that inhibition of lipid peroxidation mediated by liproxstatin-1 treatment is not sufficient to rescue SLC7A11 KO melanoma cells from death (70). Furthermore, it has also been shown that there is strong induction of lipid peroxidation during the activation of noncanonical pyroptosis that is not necessarily related to the direct execution of this death pathway (71). This evidence could limit lipid peroxidation as an exclusive hallmark of ferroptosis, driving the definition of ferroptosis toward a type of death *dependent on lipid peroxidation* (72).

## Does Targeting SLC7A11-GSH Axis in Neuroblastoma Induce Ferroptosis?

In another context and emphasizing that MYCN is a protein overexpressed in NB, recent advances have been achieved to determine that MYCN favors an increase in intracellular iron *per se*, which could favor the pharmacological sensitization of NB to ferroptosis induction (66, 67). Thus, the authors determined that inhibition of SLC7A11 with sulfasalazine (14) or inhibition of GSH synthesis with Buthionine sulfoximine (BSO) favors the induction of ferroptosis in models of NB with MYCN amplification (67). This *in vitro* evidence from patient samples is closely related to an eventual clinical application, since the toxicity of BSO has been evaluated in a phase I clinical trial, as a possible treatment for NB in conjunction with other drugs (73). Despite being relatively well tolerated, the treated patients presented vomiting/nausea as adverse effects (73). However, there is also evidence indicating that the administration of BSO can trigger kidney failure in animal models (74) and patients (75). Thus, special precautions must be taken when trying to directly extrapolate *in vitro* findings to *in vivo* models or patients.

Curiously, some of the NB cell lines used in this study show partial resistance to death induced by the SLC7A11 inhibitor and GSH depletor erastin (17), which leads to an intriguing question: why are some NB cell lines resistant to erastin but sensitive to inhibition of GSH synthesis or inhibition of SLC7A11 mediated by sulfasalazine? In this scenario, it is important to highlight that in lung adenocarcinoma cells, it was recently determined that the SLC7A11 inhibitor HG106 preferentially induces GSH depletion and cell death by apoptosis, which is inhibited by the recovery of cysteine ​​uptake, but without eventual induction of ferroptosis, since DFO treatment does not prevent cell death (76). This evidence suggests that although HG106 has the same target as erastin (SLC7A11), there are other off-targets that favor the induction of one type of death over another (apoptosis or ferroptosis) or the production of particular ROS that trigger differential cellular responses (22). Despite these pharmacological dichotomies, which induce different types of death even when the target is the same, or which have differential action mechanisms in response to treatment with SAS, erastin (IKE) or HG106, the message that remains the same: SLC7A11 is a potent therapeutic target for cancer (**Figure 2B**).

## Iron Overload as a Possible Treatment for Neuroblastoma

It was shown that NB cells with MYCN amplification are particularly sensitive to the induction of death mediated by treatment with auranofin (a rheumatoid arthritis drug) (67). Although the authors attribute the effect of auranofin to the induction of ferroptosis, treatment with Fer-1 only partially rescues cells from cell death, even when there is an increase in lipid peroxidation, and treatment with DFO effectively prevents cell death and ROS production (67). Again, this finding leads us to conclude that apparently in NBs, the lipid ROS generated are specific to this tumor type or there are parallel mechanisms of cell death, since Fer-1 is not capable of completely inhibiting cell death, even when the evidence points to iron and lipid ROS dependency. Accumulating evidence, the literature indicates that iron accumulation and increased LIP are strong candidates for exploiting the pharmacological sensitivity of high-risk NB to ferroptosis induction (**Figure 5**) (17, 50, 66, 67, 77). Thus, the use of compounds that promote the mobilization or uptake of iron in this type of tumor, in combination with ferroptosis inducers, could exploit the vulnerabilities of this tumor to favor its eradication. However, further studies are still needed to determine the potential lethal effects on nervous tissue and to assess whether these types of therapeutic agents can penetrate the blood–brain barrier.

## Glioblastoma

### Overview of Glioblastoma Treatment

Malignant gliomas are one of the most devastating and frequently diagnosed brain tumors in adults and are associated with a short life expectancy of only 12 to 15 months (78). The WHO classifies this type of tumor as grade I to IV, the latter being called glioblastoma multiforme (GBM), which corresponds to the most advanced stage and has a shorter life expectancy (78–80). The current incidence of GBM in the USA is approximately 7 per 100,000 inhabitants (79). Currently, therapy for GBM is based on surgery accompanied by radiation therapy and chemotherapy, since GBM cannot be completely removed surgically due to its infiltrative nature (78). Although radiotherapy increases the life expectancy of patients, 90% of GBMs exhibit recurrence at the original tumor site after therapy (81). Thus, all hopes for the treatment of this type of tumor are placed on the development of new agents or pharmacological strategies for successful chemotherapy. To date, the main pharmacological approaches for the treatment of GBM include the use of antiangiogenic therapies (bevacizumab, sunitinib, vandetanib), immunotherapy (anti-PD-1/PD-L1 antibodies) and various other molecular approaches, such as inhibitors of mTOR, EGFR, HSP90, and PI3K (78, 82). Unfortunately, GBMs acquire resistance to these types of treatment (78, 82). In this scenario, as a therapeutic strategy, one of the most commonly used compounds is temozolomide (TMZ), an oral alkylating agent (80, 83, 84) that targets the DNA repair enzyme O6-methylguanine DNA methyltransferase (MGMT), which has been shown to prolong the life expectancy of patients when used in conjunction with radiotherapy (84–86). Unfortunately, most GBMs recur after 2 years with cell populations resistant to this type of therapy due to stem cell properties (87–89). Based on accumulating evidence and the strong resistance of GBM to multiple therapies, the development of new drugs for the treatment of these devastating tumors is urgently needed.

### Pharmacological Ferroptosis Induction: A Therapy Against Glioblastoma?

Based on the above premise, pharmacological induction of ferroptosis could exploit the vulnerabilities of GBM cells and sensitize them to death when used in combination with other antineoplastic compounds. In line with this idea, the evidence suggests that combined treatment with ferroptosis inducers plus other antineoplastic therapies (e.g., TMZ or radiation) could lead to sensitization to this type of death in GBM cells (60, 90). This is because most GBM cells are resistant to either SLC7A11 inhibition (erastin treatment) or GPX4 inhibition (RSL-3 treatment) (54, 91, 92), although they express ACSL4 (93).

In line with this observation, high levels of SLC7A11 expression are considered to predict poor survival in patients with malignant glioma (94). At the same time, high expression of SLC7A11 is associated with epileptic seizures, stem cell properties, increased migration and invasion, neurosphere formation and increased expression of Nanog, Sox-2 and Nestin, among other proteins (95, 96). Thus, the expression of this transporter is considered a possible biomarker for the diagnosis of GBM. In this scenario, it is tempting to speculate that SLC7A11 blockade could be an excellent therapy for GBM, since its high expression level indicates a strong dependence on its function.

However, current evidence has shown that GBM cells, such as U87, U251, and U373 cells, are highly insensitive to treatment with SAS or erastin (72, 97), a phenotype that could be related to resistance mechanisms mediated by ATF4 and Nrf-2 that favor overexpression of SLC7A11 (21, 97, 98). Furthermore, the use of SAS in a clinical trial against glioma did not show a response, and various adverse effects were observed (99), which greatly complicates its future use as a ferroptosis-inducing drug in patients. It is important to note that various studies have suggested that GBM cells (and cells of other lineages) have unique sensitivity to death (theoretically ferroptotic) mediated by sorafenib treatment (97, 100). However, recently, it was shown that sorafenib failed to induce ferroptosis in a wide panel of tumor cell lines (including GBM cell lines) (72), which leads us to take special care in the interpretation and specificity of sorafenib in triggering ferroptosis.

### Molecular Pathways That Confer Resistance to Ferroptosis in Glioblastoma

In this scenario, where GBM cells show great resistance to inhibition of system $x_{\text{c}}^{-}$ , it is possible to speculate that they obtain cysteine ​​intracellularly from another source that implies mechanisms independent of the function of SLC7A11, which would explain the resistance to treatment with erastin or SAS. The main metabolic pathway that supplies cysteine ​​intracellularly in tumor cells independent of the transport activity of SLC7A11 is the transsulfuration pathway (101). The transsulfuration pathway allows methionine to be used as a substrate for cysteine ​​biosynthesis through various enzymatic reactions (101). At the same time, it has been shown that inhibition of this pathway in tumor cells makes it possible to recover sensitivity to erastin in certain cell lines other than GBM cell lines (102). Unfortunately, inhibition of the expression of cystathionine β-synthase (CBS), a key protein in the transsulfuration pathway, has been shown to promote GBM progression (103), while in other tumor models, CBS inhibition effectively causes cell death (104), which suggests that GBM cells could be resistant to ferroptosis induction, including that mediated through inhibition of system $x_{\text{c}}^{-}$ and the transsulfuration pathway.

On the other hand, one possible explanation for the strong resistance of GBM cells to the induction of ferroptosis is the protective effect exhibited by FSP1 in this type of tumor (**Figure 4**), since most GBM cells express high levels of this protein, and cotreatment with iFSP1 and RSL-3 strongly sensitizes them to ferroptosis (54, 72). However, cotreatment with erastin or SAS + iFSP1 fails to induce death in GBM cells (72). This evidence corroborates the findings that FSP1 acts independently of the GSH level (54, 55) and that it apparently can only have synergistic effects with direct GPX4 inhibitors such as RSL-3 or ML162.

Interestingly, the GPX4 depletor FIN56 (**Figure 4**) was recently shown to induce ferroptosis in *in vitro* and *in vivo* GBM models (105); this was the first study to use this compound *in vivo*. However, the trial was not carried out with tumors in nervous tissue but rather in nude mice with subcutaneous tumors, which makes it difficult to extrapolate the possible eventual effects of FIN56 on the brain, and it is not known whether this compound can cross the blood–brain barrier to be considered a potential therapy in the future.

Based on accumulating evidence and given the limitations of the use of direct GPX4 inhibitors for the treatment of tumors *in vivo*, the best therapeutic approach seems to be inhibition of SLC7A11. Along these lines, it has been demonstrated that cotreatment with IKE and radiation favors ROS production and induces cell death in GBM models (60). Concurrently, cotreatment with erastin and TMZ has been found to sensitize GBM cells to death (90). This eventual therapeutic strategy offered by treatment with SLC7A11 inhibitors should be exploited in the future in the search for compounds with synergistic activity that exploit the vulnerabilities of GBM cells.

## Conclusions and Future Perspectives

Although there are several inducers of ferroptosis, the potential use of these drugs as cancer treatments is limited because they have little bioavailability for action *in vivo*. However, with the development of IKE, an avenue was opened for ferroptosis induction as an *in vivo* treatment by targeting system $x_{\text{c}}^{-}$ (19). To date, evidence suggests that inhibition of system $x_{\text{c}}^{-}$ could be a safe therapeutic approach as a tumor suppressor. Unfortunately, several types of tumors, including NB and GBM, are resistant to system $x_{\text{c}}^{-}$ inhibition for ferroptosis induction (13, 17, 92). Thus, combination therapies of other antineoplastic drugs with IKE may represent an option for the treatment of cancers highly resistant to cell death. However, ferroptosis dogma dictates that GPX4 is the key protein (15, 106); thus, all efforts have been focused on the development of new drugs for its inhibition. Although there are direct GPX4 inhibitors, such as RSL3 (15), they have little application *in vivo* (40), and GPX4 deletion in some types of cancer is not lethal (51), suggesting that there may be other mechanisms in addition to GPX4 inhibition to suppress lethal lipid peroxidation. FSP1, GCH1 and BH4/BH2 are proteins with the ability to inhibit ferroptosis independently of GPX4 and GSH levels (54, 55, 65), and FSP1 is a *druggable* protein (54). In line with this idea, a new avenue has been opened for the development of drugs that include SLC7A11, GPX4 and FSP1 inhibitors with potential *in vivo* application as a cancer treatment.

## Author Contributions

LF, MB, KS, AG and FN conceived the ideas and concepts. LF wrote the article. AG, KS, MV and FN critically revised the manuscript. MB generated the scientific illustrations. All authors approved the final version of the manuscript. All authors contributed to the article and approved the submitted version.

## Funding

This work was supported by a Fondecyt regular grants 1181243 and 1221147, ANID PIA ECM-12 grant (to FN), Fondecyt iniciacion 11200335 (LF), Fondecyt postdoctorado 3210076 (MB) and U.S. Department of Defense W81XWH-12-1-0341 (AG).

## Conflict of Interests

The authors declare that the research was conducted in the absence of any commercial relationships that could be construed as a potential conflict of interest.

## Publisher’s Note

All claims expressed in this article are solely those of the authors and do not necessarily represent those of their affiliated organizations, or those of the publisher, the editors and the reviewers. Any product that may be evaluated in this article, or claim that may be made by its manufacturer, is not guaranteed or endorsed by the publisher.
